# Screening of Lactic Acid Bacteria Tolerant to Antimicrobial Substances and Their Effects on the Quality of *Cyperus esculentus* Silage

**DOI:** 10.3390/microorganisms13122833

**Published:** 2025-12-12

**Authors:** Hui Wang, Weiqi Liu, Shengyi Li, Shuan Jia, Xuzhe Wang

**Affiliations:** 1 College of Animal Science & Technology, Shihezi University, Shihezi 832000, China; wh17671003482@163.com (H.W.); lwq202512@163.com (W.L.); 2 Bingtuan Key Laboratory for Efficient Utilization of Non-Grain Feed Resources, Urumqi 830002, China; 3 Xinjiang Uygur Autonomous Region Animal Disease Prevention and Control Center, Urumqi 830002, China; 4 Xinjiang Uygur Autonomous Region Agricultural and Rural Reform Research Center, Urumqi 830002, China

**Keywords:** chufa, endogenous substances, metabolic pathways, fermentation quality

## Abstract

This study investigated the inhibitory effects of endogenous compounds from *Cyperus esculentus* on lactic acid bacteria and examined whether inoculation with selected strains could enhance silage fermentation quality. Lactic acid bacteria were isolated and identified from *C. esculentus* silage, and antibacterial assays were performed to screen strains suitable for inoculation. Results indicated that the endogenous compounds inhibited lactic acid bacteria, with sensitivity ranked as *Lentilactobacillus buchneri* > *Lactiplantibacillus plantarum* > *Lacticaseibacillus rhamnosus*. Scanning electron microscopy revealed no significant structural damage to bacterial cells caused by the inhibitory compounds. Metabolomic analysis suggested that these compounds may alter lactic acid bacterial metabolism by modulating key pathways, including amino acid and energy metabolism. Among the tested strains, *Lcb. rhamnosus* exhibited the highest tolerance to the endogenous compounds. Inoculation experiments demonstrated that the addition of *Lcb. rhamnosus* significantly enhanced the fermentation quality of *C. esculentus* silage.

## 1. Introduction

*Cyperus esculentus* L., commonly known as tiger nut, is an annual herbaceous plant in the family Cyperaceae [1]. Its morphology includes aerial leaves, underground tubers, and a well-developed root system. The tubers are typically spherical or ellipsoidal, with a wrinkled brown periderm, and morphologically resemble nuts. Nutrient-rich, these tubers function as the primary reproductive organs of the species [2]. Native to Africa, *C. esculentus* shows strong thermotolerance. Through long-term natural selection and artificial breeding, it has adapted to temperate and subtropical climates and shows partial tolerance to cold environments. The species is now cultivated across Southern Europe, the Americas, Africa, Australia, and China [3]. Introduced into China in 1952, *C. esculentus* has shown notable resilience to diverse abiotic stresses, including low temperatures, drought, waterlogging, poor soil fertility, and salinity. Consequently, it is often cultivated in marginal and borderland areas. Under optimal agronomic conditions, yields can reach 15–18 t·ha^−1^, but its utilization in China remains limited [4].

The leaves of *C. esculentus* are rich in carbohydrates, oils, dietary fiber, and other nutrients, containing 9.8% crude protein—comparable to that of oats. The crude fiber content reaches 19.3%, indicating suitability as a functional raw material for silage production, and crude fat content is as high as 8.9% [1,5]. Given its crude protein level comparable to that of oats, which are known for high protein content and digestibility, *C. esculentus* can be fed directly to livestock or combined with forage grasses [5]. After drying and pulverization, its stems and leaves can be incorporated into concentrated feed, reducing overall feed costs [4]. Silage technology, an efficient method for long-term preservation, enhances feed utilization; however, the adoption of *C. esculentus* in silage production remains limited. A primary constraint is the presence of endogenous inhibitory factors during processing, which compromise silage quality and stability.

The endogenous compounds of *C. esculentus* primarily comprise phenolics and flavonoids [6]. Flavonoids possess notable antibacterial activity, capable of inhibiting the growth and proliferation of beneficial microorganisms such as lactic acid bacteria, and potentially reducing their fermentation efficiency during silage. The mode of action primarily involves disrupting microbial cell membrane permeability and function, thereby inducing physiological and biochemical changes within cells, ultimately compromising fermentation quality [7]. Although previous studies have shown that flavonoids inhibit the fermentative activity of lactic acid bacteria during tiger nut silage, their precise modes of action remain unclear [8].

Currently, fundamental research on this topic remains limited both domestically and internationally. Some research teams have explored the feasibility and potential value of ensiling *C. esculentus* stems and leaves, and analyzed their chemical composition, thereby providing baseline data for investigating interactions between flavonoids and lactic acid bacteria [6]. Moreover, research on other silage crops may offer insights into the tiger nut ensiling system; however, inherent differences in plant composition and fermentation characteristics limit direct applicability of these findings, highlighting the need for targeted studies. This study employs lactic acid bacteria strains isolated from *C. esculentus* with demonstrated tolerance, to evaluate their impact on silage quality upon inoculation, and to identify effective approaches for enhancing the value of tiger nut in livestock production.

## 2. Materials and Methods

### 2.1. Harvesting and Silage Production of C. esculentus

The silage used in this experiment was prepared from *C. esculentus* stems and leaves harvested from Plot No. 6 in the plantation area of the No. 54 Regiment, Third Division, Xinjiang Production and Construction Corps. The growth period extended from 27 May to 7 October. On 4 October 2023, stems and leaves were harvested by cutting at a height of 3–4 cm above the ground surface. The material was chopped into 2–3 cm segments and packed into transparent plastic bags (500 ± 10 g per bag). Each bag was heat-sealed, vacuum-packed, and stored under anaerobic conditions at 18–23 °C. After 60 days of fermentation, the silage was retained for use in the trial.

### 2.2. Analysis of Culture-Based Microbial

Eight well-preserved silage bags were selected from *C. esculentus* stem and leaf silage samples fermented for 60 days. Approximately 10 g of material from each bag was transferred into a sterile Erlenmeyer flask containing 90 mL of sterile diluent and incubated at 37 °C with shaking at 180 rpm for 2 h. After sedimentation, the supernatant was collected and serially diluted to 10^−4^, 10^−5^, and 10^−6^. Aliquots from each dilution were inoculated onto MRS and M17 agar plates, with two replicates for each medium–dilution combination, and incubated anaerobically at 37 °C for 2 days. Colonies with distinct morphologies were subjected to catalase tests and Gram staining. Eight isolates exhibiting typical lactic acid bacteria (LAB) characteristics were obtained: strains M1–M4 from MRS and M5–M8 from M17.

LAB isolates were purified on respective media using the four-quadrant streak method for 2–3 successive subcultures. Pure cultures were grown in the corresponding liquid media anaerobically for 1 day, mixed with an equal volume of 50% glycerol, dispensed into cryovials, and stored at −80 °C. Purified strains were sent to RuiBoXingKe Biotechnology Co., Ltd. (Beijing, China) for 16S rDNA sequencing using universal bacterial primers 27F (5′-GAGTTCTCGGAGTCACGAAGAGTTTGATCCTGGC-3′) and 1495R (5′-GGATCACTTCACACAGGACTACGGGTACCTTGTT-3′).

The 16S rDNA sequences were assembled, quality-trimmed, and submitted to the NCBI GenBank database. Sequence homology analysis was conducted using BLASTn (NCBI BLAST+ v2.14.1). Sequences of type strains with highest similarity to the tested isolates were downloaded as references. Multiple sequence alignment was performed using MEGA version 11.0. A phylogenetic tree was constructed with the Neighbor-Joining method based on alignment results, and bootstrap analysis with 1000 replications was performed to evaluate tree topology reliability. Taxonomic status was determined according to clustering positions and sequence similarity.

### 2.3. Assay of the Inhibitory Activity of C. esculentus Extract Against LAB

Fresh, healthy stems and leaves of *C. esculentus* were selected, with withered leaves, impurities, and soil removed. The materials were rinsed twice with tap water, followed by three washes with distilled water, and placed on clean filter paper to remove surface moisture. Processed stems and leaves were cut into 2–3 cm segments and dried to constant weight in a hot-air oven at 60 °C. Dried samples were ground into a fine powder using a grinder and passed through a 40-mesh sieve for subsequent use.

A 5 g portion of powder was placed in a stoppered Erlenmeyer flask, and 200 mL of 70% (*v*/*v*) ethanol was added as the extraction solvent. Ultrasonic extraction was performed at room temperature for 30 min. The mixture was first filtered through sterile gauze, then through qualitative filter paper to obtain the filtrate. The filtrate was transferred to a rotary evaporation flask and concentrated under vacuum at 50 °C in a water bath until a viscous extract was obtained. The concentrated extract was placed in sterile centrifuge tubes and stored at 4 °C until antimicrobial assays.

The antibacterial activity of *C. esculentus* leaf extract against different LAB strains was evaluated using the Oxford cup method. Pre-activated LAB were cultured in MRS liquid medium at 37 °C for 16–18 h to reach the logarithmic phase. An appropriate volume of bacterial suspension was added to melted MRS agar (upper layer, 4–5 mL per dish) at ~45 °C and overlaid onto solidified lower-layer MRS agar to prepare double-layer plates, ensuring uniform bacterial distribution. A stainless-steel Oxford cup (inner diameter ~6 mm, height~10 mm) was placed vertically on the agar surface using sterile tweezers, ensuring close contact to prevent leakage. Pre-prepared, sterilized *C. esculentus* leaf extract was added to each cup (0.25 mL per cup) with treatments of 50, 100, 150, 200, and 250 μL. Plates were placed in anaerobic jars and incubated at 37 °C for 24 h. After incubation, the diameter of the transparent inhibition zone around each cup was measured using a vernier caliper. Sensitivity was classified as resistant (<10 mm), moderately sensitive (10–15 mm), or highly sensitive (>15 mm) based on inhibition zone diameter [9].

### 2.4. Assay of the Inhibitory Activity of Endogenous Substances from C. esculentus Extract Against LAB

To further investigate the inhibitory mechanism of *C. esculentus* extracts on LAB growth, and based on previous research on endogenous antimicrobial substances, two representative flavonoids—naringenin and diosmetin—were selected for analysis. Curcumin, a common phenolic antimicrobial agent, was used as a positive control [10,11].

Naringenin and diosmetin contents in *C. esculentus* extracts were determined using HPLC with a ZORBAX SB-C18 reversed-phase column. The mobile phase comprised 0.1% aqueous formic acid (solvent A) and acetonitrile (solvent B), with a flow rate of 1.0 mL/min, column temperature 30 °C, injection volume 10 μL, and detection wavelength 280 nm. Gradient elution: 0–10 min, 15–40% B; 10–20 min, 40–60% B; 20–25 min, 60–90% B; 25–30 min, 90–15% B, followed by 5 min column re-equilibration. Standard and sample solutions were prepared following the external standard method. Naringenin and diosmetin standards (1.0 mg/mL) were prepared in chromatographic-grade methanol, mixed and serially diluted to 2, 5, 10, 20, and 50 μg/mL, filtered through 0.22 μm organic membranes, and analyzed from low to high concentration. Calibration curves were generated by plotting mass concentration versus peak area, and linear regression equations were obtained. *C. esculentus* extract samples were redissolved in methanol by ultrasonication, filtered through 0.22 μm membranes, and analyzed. Target peaks were identified by retention time, and concentrations calculated from calibration equations.

Based on quantified endogenous substances, concentration gradients were set as: Curcumin: 1, 4, 8, 16 μg/mL; Naringenin: 0.5, 1, 4, 8 μg/mL; Diosmetin: 0.5, 1, 3, 6 μg/mL. Antibacterial activity was assessed by paper disk diffusion. Sterilized paper disks (6 mm diameter) were immersed in test solutions for 24 h, air-dried, and placed on MRS plates spread with 200 μL LAB suspension in zoned areas according to concentration. Sterile water served as the blank control; each treatment was performed in triplicate. Plates were incubated anaerobically at 37 °C for 24 h, and inhibition zone diameters measured. Sensitivity interpretation: >15 mm, high; 10–15 mm, moderate; 6–10 mm, low; <6 mm, none [12].

### 2.5. Morphological Observation of Bacteria

Based on inhibition zone assay results, the optimal inhibitory concentration of each endogenous substance for each bacterial strain was determined. A 50 μL LAB suspension was inoculated into 40 mL MRS liquid medium in culture flasks, and the corresponding optimal concentration of the endogenous substance was added. Cultures were incubated at 37 °C with shaking (180 rpm) for 24 h.

A 1.5 mL aliquot of cultured bacterial suspension was centrifuged at 12,000× *g* for 10 min at 4 °C, and the supernatant discarded. A glass slide was placed into the tube containing the bacterial pellet, and 2.5% glutaraldehyde was added to fully cover the slide for fixation at 4 °C for 12 h. Samples were rinsed and immersed in 0.1% phosphate-buffered solution (PBS) at room temperature three times, each for 15 min. Dehydration was conducted using an ethanol gradient (30%, 50%, 70%, 80%, 90%, and 100%), with each concentration applied for 15 min at room temperature; the 100% ethanol step was repeated twice to ensure complete dehydration.

Dehydrated samples were dried using a critical point dryer with CO_2_ as the transitional medium. After drying, samples were mounted on SEM stubs and sputter-coated with gold (~10 nm thickness) to improve conductivity. Prepared samples were stored under dry conditions, then examined and imaged using a field-emission scanning electron microscope (FE-SEM).

### 2.6. LC-MS/MS Analysis

Based on antibacterial assay results, diosmetin—the most effective compound—was selected for non-targeted metabolomics analysis, with curcumin as the positive control. LAB in the logarithmic phase were inoculated into fresh MRS broth at 1% (*v*/*v*). Diosmetin was used at 6 μg/mL, based on its quantified content in tiger nut extracts. Curcumin was used at 16 μg/mL, experimentally shown to have antibacterial activity equivalent to 6 μg/mL diosmetin. MRS broth without added compounds served as the blank control. All groups were incubated anaerobically at 37 °C with constant shaking for 24 h.

Immediately after incubation, all procedures were conducted on ice. Cultures were centrifuged at 12,000× *g* for 10 min at 4 °C, and supernatants discarded. Pellets were washed twice with ice-cold PBS and quenched in liquid nitrogen to terminate metabolic activity. Frozen, disrupted pellets were extracted with methanol/water (80:20, *v*/*v*), vortexed, sonicated, and centrifuged at low temperature to remove debris. Supernatants were collected for LC-MS analysis.

Non-targeted metabolomics was performed using UHPLC (Thermo Scientific, Waltham, MA, USA) coupled to a Q Exactive Orbitrap mass spectrometer (Thermo Scientific, Waltham, MA, USA). Chromatographic separation was achieved on a BEH C18 column (100 mm × 2.1 mm, 1.7 µm) with mobile phase: 2% aqueous acetonitrile with 0.1% formic acid (A) and acetonitrile with 0.1% formic acid (B). Column temperature: 40 °C; injection volume: 3 μL; flow rate: 0.40 mL/min.

Mass spectrometry was performed by electrospray ionization (ESI) in positive and negative ion modes. Scan range: *m*/*z* 50–1200. Source parameters: spray voltage, 3.5 kV (positive) and −3.0 kV (negative); sheath gas 50 psi; auxiliary gas 13 psi; heater temperature 450 °C. MS/MS was conducted with stepped normalized collision energy at 20, 40, and 60 V.

### 2.7. Effects of LAB Addition on the Quality of C. esculentus Stems and Leaves Silage

To evaluate the effect of LAB addition on *C. esculentus* leaf silage quality, two treatments were established: a control sprayed with sterile water and an experimental group inoculated with *Lacticaseibacillus rhamnosus* at 5 × 10^7^ CFU/g fresh matter, each with five replicates. *Lcb. rhamnosus* was cultured in MRS broth, and cell density was determined by the plate count method prior to inoculation. The bacterial suspension or sterile water was evenly sprayed onto pre-crushed *C. esculentus* stems and leaves using sterile spray bottles, followed by thorough mixing. For each treatment, 500 g samples were placed in transparent polyethylene one-way valve bags (40 cm × 30 cm × 0.24 mm), vacuum-sealed, and fermented at ambient temperature (20 ± 3 °C) for 60 days before analysis.

On day 60, bags were opened and samples collected for nutritional composition analysis. Dry matter (DM) was determined by oven drying at 105 °C; crude protein (CP) was measured using the Kjeldahl method; water-soluble carbohydrates (WSC) by the anthrone colorimetric method; neutral detergent fiber (NDF) and acid detergent fiber (ADF) by the Van Soest method; and ash content by incineration at 550 °C.

For fermentation characteristics, pH was measured with a pH meter (pHs-3c, LeiCi); volatile fatty acids (lactic acid, LA, and acetic acid, AA) were quantified by high-performance liquid chromatography (HPLC); and ammonia nitrogen (NH_3_-N) was determined using the phenol–hypochlorite colorimetric method.

Aerobic stability was assessed by disinfecting bag openings before sampling to reduce contamination and moisture loss. A multi-probe temperature recorder was used to monitor both sample temperature (probe at bag center) and ambient temperature (three external probes). Aerobic stability time (Ast) was defined as the point when sample temperature exceeded ambient temperature by 2 °C, indicating the onset of silage spoilage.

### 2.8. Statistical Analysis

Raw experimental data were organized and summarized using WPS Office. Basic statistical analyses were performed in SPSS version 27.0. Two-way ANOVA (factorial design) was used to evaluate the main effects of experimental factors and their interactions, while one-way ANOVA assessed the effects of single factors on each parameter. Multiple comparisons were conducted using Duncan’s post hoc test, with significance set at *p* < 0.05. Silage data were analyzed with independent-samples *t*-tests to determine differences between control and experimental groups, with significance defined as *p* < 0.05.

For non-targeted metabolomics, multivariate statistical analyses were conducted in R 4.5.2, and volcano plots of differential metabolites were generated. Metabolic pathway enrichment analysis was implemented using Python 3.12.x scripts. Statistical charts and visualizations were created in Origin version 2024.

## 3. Results

### 3.1. Morphological Characteristics and Gene Sequences of LAB Strains

**Figure 1:**
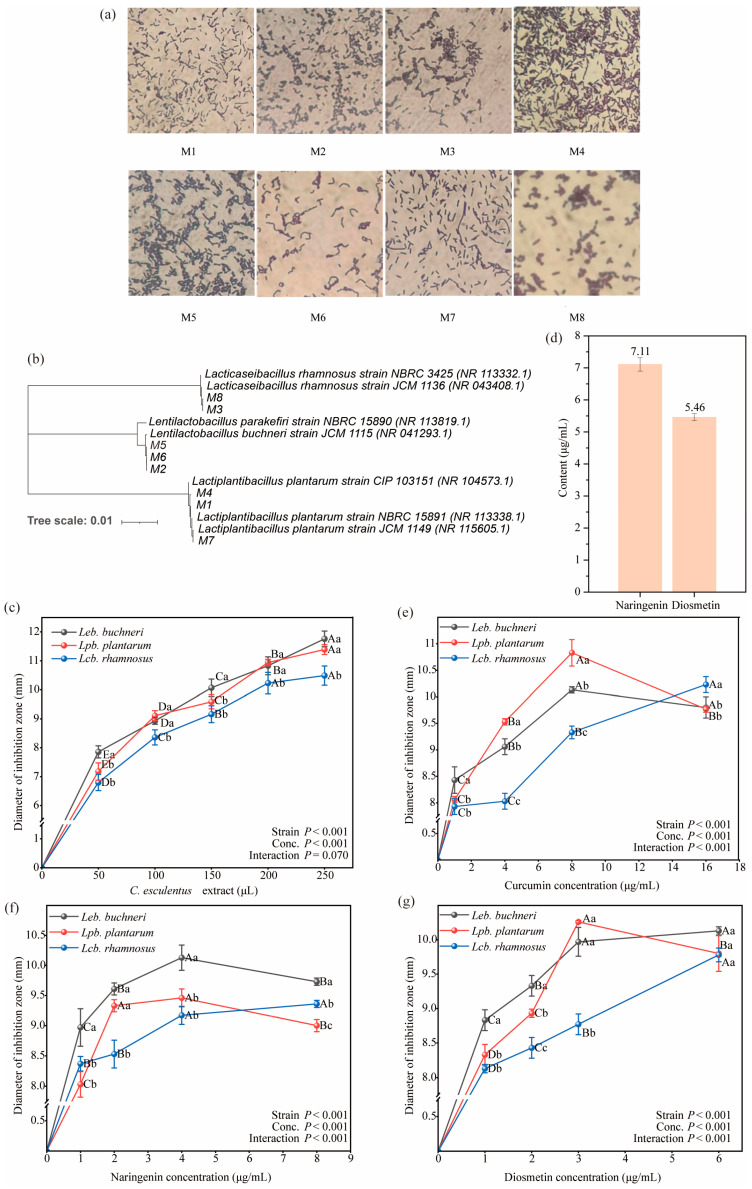
Identification of lactic acid bacteria (LAB) and the antibacterial effects of tiger nut extracts and their active components. (**a**) Micrographs of the eight screened LAB strains. (**b**) Phylogenetic tree of the eight LAB strains constructed based on gene sequences. (**c**) Effects of tiger nut extracts on the inhibition zone diameters of three LAB strains. (**d**) Content analysis of naringenin and diosmetin among the endogenous substances in tiger nuts. (**e**–**g**) Effects of treatments with curcumin (**e**), naringenin (**f**), and diosmetin (**g**) on the inhibition zone diameters of three LAB strains. Data are presented as means ± standard error (SE). Different uppercase letters indicate significant differences among different concentrations within the same strain (*p* < 0.05); different lowercase letters indicate significant differences among different strains at the same concentration (*p* < 0.05).

Microscopic examination and colony morphology analysis (Figure 1a) resulted in the isolation of eight LAB strains. Colonies of strains No. 1, No. 4, and No. 7 were large, regular in outline, with smooth and fine surfaces, cream coloration, uniform texture, and opaque appearance. Microscopy revealed regular rod-shaped, non-spore-forming cells, consistent with the morphological characteristics of the family *Lactobacillaceae*. Strains No. 3 and No. 8 formed medium-sized colonies with smooth and fine surfaces, grey-white and opaque coloration, and slightly raised profiles; microscopic observation showed short rod-shaped, non-spore-forming cells, also indicative of typical *Lactobacillaceae* morphology. Strains No. 2, No. 5, and No. 6 exhibited small colonies with smooth and fine surfaces, milky-white semi-transparent coloration, and no spore formation; microscopy revealed single rods or chain-arranged cells, likewise conforming to *Lactobacillaceae* morphological criteria. Catalase assays demonstrated uniformly negative reactions for all strains, with no bubble formation, while Gram staining consistently yielded purple coloration, confirming that all isolates were Gram-positive rods (Table 1). Sequencing of the eight isolates from *C. esculentus* silage identified strains No. 3 and No. 8 as *Lacticaseibacillus rhamnosus*, strains No. 2, No. 5, and No. 6 as *Lentilactobacillus buchneri*, and strains No. 1, No. 4, and No. 7 as *Lactiplantibacillus plantarum* (Table 2). A phylogenetic tree was subsequently constructed based on sequencing data to depict the evolutionary relationships between the isolated strains and reference sequences (Figure 1b).

Note: In the figure, “*Leb. buchneri*” refers to *Lentilactobacillus buchneri*, “*Lpb. plantarum*” refers to *Lactiplantibacillus plantarum*, and “*Lcb. rhamnosus*” refers to *Lacticaseibacillus rhamnosus*. “Strain *p*” and “Conc. *p*” represent the *p*-values for the main effects of LAB species and compound concentration, respectively, while “Interaction *p*” denotes the *p*-value for the interaction effect between them. The same applies hereinafter.

### 3.2. Inhibitory Effect of C. esculentus Extract on the Growth of LAB

**Figure 2:**
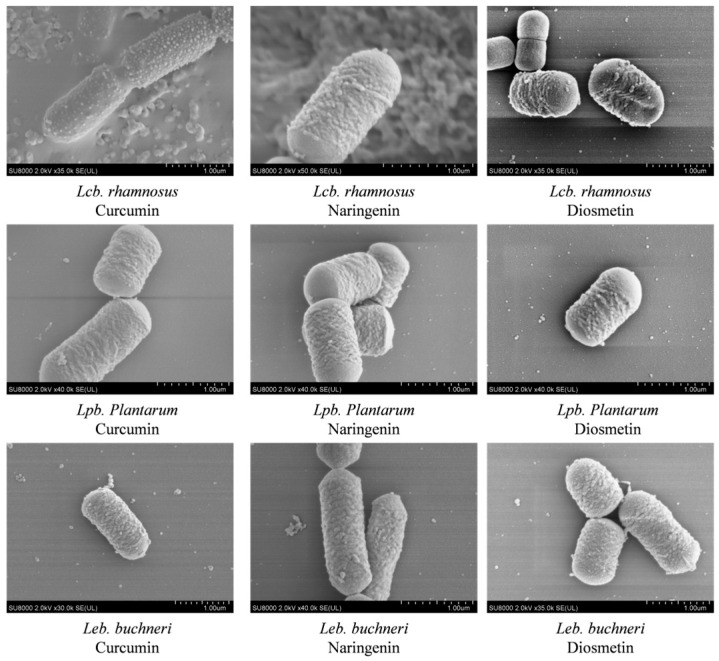
Electron microscopic observation of the effects of curcumin, naringenin, and diosmetin on three LAB strains.

Figure 2 illustrates the inhibitory effects of varying concentrations of *C. esculentus* extract on three LAB strains. Increasing extract volume was associated with a progressive enlargement of inhibition zones for all tested strains. At a low dosage (50 μL), *Leb. buchneri* exhibited the largest inhibition zone. Upon increasing the dosage to 100 μL, inhibition zones for all strains expanded; differences between *Leb. buchneri* and *Lpb. plantarum* were statistically insignificant, whereas *Lcb. rhamnosus* remained notably less affected. Further increases to 150 μL resulted in continued inhibition zone enlargement, accompanied by a reduction in interstrain variability. At higher dosages (200–250 μL), *Leb. buchneri* and *Lpb. plantarum* demonstrated comparable inhibition diameters, both substantially exceeding those of *Lcb. rhamnosus*. Collectively, the results indicate that the antibacterial activity of *C. esculentus* extract is concentration-dependent, with strain sensitivity ranking as *Leb. buchneri* > *Lpb. plantarum* > *Lcb. rhamnosus*.

### 3.3. Inhibitory Effect of Endogenous Substances in C. esculentus Extract on LAB

The *C. esculentus* extract contained 7.11 μg/mL naringenin and 5.46 μg/mL diosmetin (Figure 1d). As illustrated in Figure 1e–g, curcumin, naringenin, and diosmetin each exhibited inhibitory activity against *Leb. buchneri*, *Lcb. rhamnosus*, and *Lpb. plantarum*. Across the four tested concentration gradients, most inhibition zone diameters were < 10 mm, indicative of mild sensitivity, while a smaller proportion ranged between 10 mm and 14 mm, corresponding to moderate sensitivity.

In the curcumin assay, *Leb. buchneri* exhibited the largest inhibition zone at 1 μg/mL, *Lpb. plantarum* at 4 μg/mL and 8 μg/mL, and *Lcb. rhamnosus* at 16 μg/mL. Comparative analysis revealed that curcumin at 16 μg/mL produced the highest inhibition for *Lcb. rhamnosus*, whereas 8 μg/mL was optimal for *Leb. buchneri* and *Lpb. plantarum*.

In the naringenin assay, *Leb. buchneri* consistently presented the largest inhibition zones at equal concentrations, indicating strongest susceptibility, while *Lcb. rhamnosus* and *Lpb. plantarum* showed smaller zones. The most effective inhibitory concentration was 4 μg/mL for *Leb. buchneri* and *Lpb. plantarum*, and 8 μg/mL for *Lcb. rhamnosus*.

In the diosmetin assay, inhibition zone diameters at 1, 2, and 6 μg/mL followed the order *Leb. buchneri* > *Lpb. plantarum* > *Lcb. rhamnosus*, while at 3 μg/mL, *Lpb. plantarum* exhibited the largest zone. The optimal inhibitory concentration was 6 μg/mL for *Lpb. plantarum* and 3 μg/mL for both *Leb. buchneri* and *Lcb. rhamnosus*.

Overall, *Lcb. rhamnosus* displayed the highest tolerance to *C. esculentus*-derived compounds, whereas *Leb. buchneri* was the most sensitive. Curcumin and diosmetin exerted relatively strong inhibitory effects across lactic acid bacteria, with both demonstrating the most pronounced activity among the tested metabolites.

### 3.4. Observation of the Morphological Changes in LAB Induced by Different Endogenous Substances from C. esculentus Extract

As shown in Figure 2, the addition of various test substances did not produce noticeable morphological damage in the LAB cells. Under treatments with the two endogenous inhibitors and curcumin, the cellular structures and morphologies of the three LAB strains displayed only minor alterations and generally remained intact. The bacterial cells preserved their characteristic rod-shaped form, with no substantial variation in cell length. Overall, the cells appeared structurally robust and turgid, with membranes largely complete and only slight surface roughness observed. These results indicate that endogenous compounds from tiger nut do not induce marked morphological disruption in LAB cells, and their inhibitory effects are therefore unlikely to be associated with morphological alterations.

### 3.5. Metabolomics Analysis of the Mechanisms by Which C. esculentus Endogenous Substances Affect LAB

**Figure 3:**
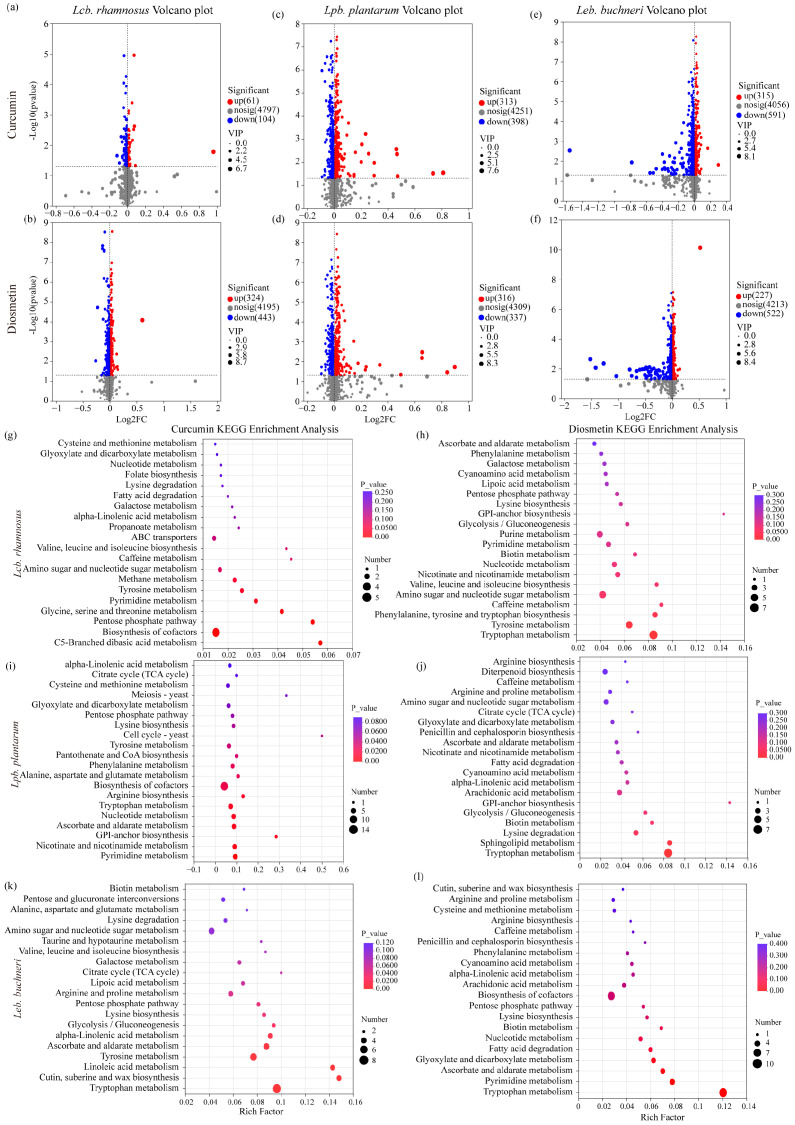
Differential metabolite analysis and KEGG pathway enrichment of three lactic acid bacteria strains treated with curcumin and diosmetin. (**a**,**b**) Volcano plots of *L. rhamnosus* treated with curcumin and diosmetin, respectively; (**c**,**d**) Volcano plots of *Lpb. plantarum* treated with curcumin and diosmetin, respectively; (**e**,**f**) Volcano plots of *L. buchneri* treated with curcumin and diosmetin, respectively. (**g**,**h**) KEGG pathway enrichment of *L. rhamnosus* treated with curcumin and diosmetin, respectively; (**i**,**j**) KEGG pathway enrichment of *Lpb. plantarum* treated with curcumin and diosmetin, respectively; (**k**,**l**) KEGG pathway enrichment of *L. buchneri* treated with curcumin and diosmetin, respectively.

The metabolic responses of the three LAB strains to curcumin and diosmetin, relative to the control group, are presented in Figure 3a–f.

For *Lcb. rhamnosus* (Figure 3a,b), the two compounds elicited markedly different effects. In the curcumin-treated group, metabolic alterations were minimal, with only 61 significantly upregulated and 104 downregulated metabolites among 4797 differential metabolites. In contrast, the diosmetin-treated group exhibited pronounced changes, encompassing 4195 differential metabolites, of which 324 were significantly upregulated and 443 downregulated. Overall, this strain demonstrated weak responsiveness to both compounds, with downregulation occurring slightly more frequently.

For *Lpb. plantarum* (Figure 3c,d), the curcumin-treated group contained 4251 differential metabolites, including 313 significantly upregulated and 398 downregulated. In the diosmetin-treated group, 4309 differential metabolites were identified, with 316 significantly upregulated and 337 downregulated. Curcumin treatment predominantly resulted in upregulation, whereas diosmetin induced a bidirectional regulatory effect, with downregulation being more prevalent.

For *Leb. buchneri* (Figure 3e,f), 4056 differential metabolites were identified under curcumin treatment, with 315 significantly upregulated and 591 downregulated. Diosmetin treatment yielded 4213 differential metabolites, comprising 227 significantly upregulated and 522 downregulated. Compared to curcumin, diosmetin caused more extensive metabolic alterations, with a higher number of downregulated metabolites, indicating a stronger inhibitory effect.

In summary, *Leb. buchneri* exhibited greater sensitivity to diosmetin than to curcumin, showing extensive upregulation alongside substantial downregulation. *Lpb. plantarum* displayed predominantly upregulated metabolites under curcumin treatment, whereas diosmetin produced a bidirectional regulatory response with more frequent downregulation. *Lcb. rhamnosus* showed high tolerance to both compounds, with only limited changes in metabolite profiles, predominantly mild downregulation.

Figure 3g–l presents the KEGG metabolic pathway enrichment analysis of differential metabolites in the three LAB strains following treatment with curcumin and diosmetin.

Both compounds significantly influenced multiple metabolic pathways across all strains, with several common alterations observed. The shared affected pathways primarily encompassed diverse amino acid metabolism routes (including cysteine and methionine metabolism, aspartate and glutamate metabolism, arginine and proline metabolism, and tryptophan metabolism), central energy metabolism pathways (such as the citric acid cycle, glyoxylate and dicarboxylate metabolism, and the pentose phosphate pathway), as well as coenzyme and nucleotide metabolism (including pantothenate and CoA biosynthesis, nicotinate and nicotinamide metabolism, and pyrimidine metabolism).

In addition, strain-specific responses to each compound were evident:

In *Lcb. rhamnosus* (Figure 3g,h), curcumin uniquely affected glycine, serine, and threonine metabolism and C5-branched dibasic acid metabolism; diosmetin specifically influenced phenylalanine, tyrosine, and tryptophan biosynthesis, ascorbate metabolism, and lysine biosynthesis.

In *Lpb. Plantarum* (Figure 3i,j), curcumin uniquely enriched biotin metabolism, branched-chain amino acid biosynthesis, and cell cycle–related pathways; diosmetin specifically modulated sphingolipid metabolism, polysaccharide and nucleotide sugar metabolism, and transmembrane transport systems (ABC transporters).

In *Leb. Buchneri* (Figure 3k,l), curcumin uniquely enriched the longevity-regulating pathway and the glycosylphosphatidylinositol (GPI)-anchor biosynthesis pathway associated with membrane structure, whereas diosmetin specifically impacted antioxidant-related pathways, including ascorbate and aldarate metabolism, sulfur metabolism, and ubiquinone biosynthesis.

Overall, the metabolic regulation of LAB by curcumin and diosmetin followed a dual pattern characterized by a “shared core pathway impact + strain-specific modulation.” The shared impact involved universal regulation of amino acid, energy, and nucleotide metabolism, while strain-specific modulation reflected the activation or inhibition of distinct functional pathways, highlighting differences in strain tolerance and the underlying mechanisms of action of these compounds.

### 3.6. Analysis of LAB Addition on C. esculentus Silage Quality

#### 3.6.1. Impact of *Lpb. Plantarum* on *C. esculentus* Silage Fermentation Quality

Table 3 illustrates the variation in DM, CP, NDF, ADF, WSC, and ash content of *C. esculentus* straw silage under treatments without LAB additives and with *Lcb. rhamnosus* supplementation. After 60 days of fermentation, DM and CP contents in the treatment group were significantly higher than those in the control group. At the same time point, NDF content in the control group was significantly lower than in the treatment group, whereas ADF content showed no significant difference between groups. Additionally, WSC content was markedly higher in the treatment group compared to the control, while ash content did not differ significantly between treatments.

#### 3.6.2. Effects of *Lcb. Rhamnosus* on Fermentation Quality and Aerobic Stability of *C. esculentus* Silage

Table 4 depicts the changes in pH, NH_3_-N, LA, AA, and aerobic stability of *C. esculentus* straw silage under treatments without LAB additives and with *Lcb. rhamnosus* supplementation. After 60 days of fermentation, the pH in the treatment group decreased to 4.76, significantly lower than that of the control group (6.55). The NH_3_-N content was markedly higher in the control group compared to the treatment group. In contrast, LA and AA contents in the treatment group were significantly elevated relative to the control. Aerobic stability of the treatment group was approximately 121 h, compared to 72 h in the control, representing a significant extension of about 49 h.

## 4. Discussion

### 4.1. Effects if Endogenous Compounds from C. esculentus on the Growth and Morphology of LAB

The *C. esculentus* extract exhibited a pronounced inhibitory effect on all three LAB strains, with inhibition zone diameters increasing significantly as extract volume increased, indicating a typical concentration-dependent pattern. These findings suggest that tiger nut extract may contain antimicrobial active compounds whose activity is concentration-dependent, and that higher doses can more effectively suppress LAB growth.

Under low-dose conditions (50 μL), the inhibition zone of *Leb. buchneri* was significantly larger than that of *Lpb. plantarum* and *Lcb. rhamnosus* (*p* = 0.021), indicating greater sensitivity to the active components of *C. esculentus*. This difference may be associated with variations in cell wall structure, membrane lipid composition, and metabolic tolerance among strains [13]. *Leb. buchneri* may lack certain defense mechanisms against plant-derived antimicrobial substances (such as phenolics and polyphenols), and therefore can display a relatively large inhibition zone even at low concentrations [14]. Conversely, the inhibition zones of *Lcb. rhamnosus* were significantly smaller than those of the other two strains across all tested doses, suggesting stronger tolerance to tiger nut extract. This tolerance may be linked to a more stable membrane lipid structure or the presence of more efficient efflux systems [15].

As the volume of *C. esculentus* extract increased to 100–150 μL, the inhibition zones of all three strains expanded and strain differences diminished, indicating that high concentrations of active compounds exert a relatively uniform inhibitory effect on different LAB species. At high doses (200–250 μL), inhibition zone diameters of *Leb. buchneri* and *Lpb. plantarum* were similar, but still significantly larger than those of *Lcb. rhamnosus*. This suggests that at elevated concentrations, tiger nut extract can overcome the defense mechanisms of moderately tolerant strains, yet differences in inhibition remain for highly tolerant strains.

Previous studies have reported that *C. esculentus* contains a variety of bioactive compounds, including phenolic acids, flavonoids, polyphenols, essential oils, and fatty acids. These constituents can disrupt bacterial membrane structures, interfere with protein synthesis, or inhibit energy metabolism [10]. In addition, the unsaturated fatty acids present in tiger nut may increase membrane permeability, leading to leakage of intracellular contents and consequently promoting inhibition zone formation. Differences in sensitivity among LAB strains to these components may be related to variations in membrane lipid composition, cell wall thickness, and mechanisms of acid and oxidative stress resistance [16].

Among these compounds, naringenin and diosmetin are representative flavonoid antibacterial substances in *C. esculentus* extracts, while curcumin is a common plant endogenous antibacterial agent. Their inhibitory effects on different LAB species exhibited distinct concentration dependence and strain specificity. Naringenin showed its strongest inhibition against *Leb. buchneri* at 4 μg/mL, with an inhibition zone diameter of 10.13 mm. Curcumin’s inhibitory effect on *Lpb. plantarum* increased steadily with concentration, reaching 10.83 mm at 8 μg/mL. Resveratrol displayed a “low-concentration enhancement, high-concentration attenuation” phenomenon against *Leb. buchneri*: at 0.25 mg/mL, the inhibition zone was 9.37 mm, whereas at 2 mg/mL it decreased to 8.23 mm. This concentration-dependent pattern may be related to saturation of transmembrane transport within bacterial cells and the binding affinity of the compound to its molecular targets [17]. For example, flavonoids and polyphenols can exert antibacterial activity by inhibiting bacterial respiratory chain enzyme systems [18]. At high concentrations, target saturation or induction of bacterial adaptive responses may attenuate antimicrobial efficacy [19]. Marked strain-specific differences were also observed: *Lpb. plantarum* was relatively sensitive to curcumin and diosmetin; *Leb. buchneri* responded more strongly to naringenin and low concentrations of resveratrol; and *Lcb. rhamnosus* reacted strongly to high concentrations of curcumin. Such variations in sensitivity may stem from diversity in metabolic pathways [20].

This hypothesis is supported by morphological observations. SEM revealed that after treatment with naringenin, curcumin, diosmetin, and resveratrol, the cell structures of *Lcb. rhamnosus*, *Leb. buchneri*, and *Lpb. plantarum* remained intact, with no signs of lysis, fragmentation, or collapse of the cell wall. Severe damage was not observed; only a subset of cells displayed ultrastructural changes such as surface roughness and fine wrinkles. This indicates that the inhibitory effects of *C. esculentus* endogenous compounds on LAB are unlikely to be mediated by direct physical damage, but instead via indirect mechanisms involving membrane functionality, metabolic pathways, or quorum sensing [21].

Highly hydrophobic flavonoids, such as naringenin and curcumin, can embed within the phospholipid bilayer of the cell membrane, altering membrane fluidity and permeability, thereby affecting transport processes and the activity of membrane-associated enzymes [22]. The concentration-dependent peak inhibition of *Leb. buchneri* observed with naringenin may be related to its interference with key glycolytic enzymes or respiratory chain complexes [23]. Curcumin may indirectly exert inhibitory effects by disrupting fatty acid synthesis or enzymes involved in DNA replication [24]. Moreover, these compounds can interfere with quorum sensing systems, thereby inhibiting biofilm formation and bacterial cooperation [25]. Similar mechanisms have been reported for other plant-derived antimicrobials: Cox et al. [26] found that *Melaleuca alternifolia* essential oil caused potassium ion leakage, had a greater effect in *E. coli*, inhibited bacterial respiration, and increased membrane permeability as indicated by propidium iodide uptake. Ogunremi et al. [27] reported that *C. esculentus* extracts deplete branched-chain amino acids in LAB, triggering metabolic arrest without affecting cell wall synthesis.

Collectively, these findings support the concept that “morphological integrity and physiological function can be dissociated,” suggesting that interactions between *C. esculentus* endogenous compounds and LAB should be explored beyond the classical framework of morphological damage, with greater emphasis on deeper mechanisms such as metabolic inhibition.

### 4.2. Effects of Different Endogenous Substances on LAB Metabolism

Metabolomic analysis revealed pronounced strain-specific differences in the metabolic regulation of LAB in response to curcumin and diosmetin. These differences were evident not only in the overall distribution shifts of metabolic features, but also in the number of differential metabolites and the types of metabolic pathways affected. Overall, diosmetin had a stronger metabolic impact on *Leb. buchneri* and *Lpb. plantarum* than curcumin, whereas *Lcb. rhamnosus* exhibited high tolerance to both compounds, maintaining metabolic homeostasis relatively well after treatment.

From the perspective of overall metabolic profiles, PCA analysis suggested distinct mechanisms for the two compounds. In certain strains—particularly *Lpb. plantarum*—the metabolic features in curcumin-treated cells nearly overlapped with those of the control, indicating limited regulatory effects. In contrast, diosmetin induced a pronounced shift in the principal components of *Leb. buchneri* and *Lpb. plantarum*, implying stronger regulation or even inhibition within energy conversion, biosynthetic, and catabolic pathways. Such differences may stem from the high membrane-affinity potential of diosmetin or its direct action on key enzyme systems involved in antioxidant defense and carbon metabolism [28].

Statistical analysis of differential metabolites further supports this hypothesis. In sensitive strains, diosmetin triggered a higher number of downregulated metabolites—particularly in *Leb. buchneri*, where the count was markedly greater than in curcumin-treated cells. This suggests that diosmetin may exert systemic inhibition on pathways linked to membrane structure, antioxidant systems, and biosynthesis of specific amino acids [29]. In *Lpb. plantarum*, both compounds triggered bidirectional regulation; however, curcumin more frequently promoted upregulation, whereas diosmetin tended toward downregulation, indicating distinct effects on the metabolic balance of the strain. For *Lcb. rhamnosus*, both compounds induced relatively few and low-magnitude changes, reflecting strong metabolic buffering or tolerance capacity and resistance to disruption by exogenous bioactive compounds.

KEGG enrichment analysis revealed two distinct regulatory patterns: common and strain-specific. Commonly affected fundamental pathways included multiple amino acid metabolic processes (e.g., cysteine and methionine metabolism, arginine and proline metabolism), core energy metabolism (tricarboxylic acid cycle, glyoxylate and dicarboxylate metabolism), and coenzyme/nucleotide metabolism (pantothenate and CoA biosynthesis, niacin metabolism). This broad involvement in core pathways indicates that both compounds primarily target fundamental processes in LAB metabolism. Notably, antibacterial mechanisms targeting such core metabolic processes have also been described for other natural active agents [30]. In contrast, strain-specific pathways more clearly reflected unique compound–strain interactions. For example, in *Leb. buchneri*, diosmetin additionally modulated ascorbate and sulfur metabolism; in *Lpb. plantarum*, it interfered with sphingolipid and polysaccharide metabolism; and in *Lcb. rhamnosus*, it significantly affected phenylalanine/tyrosine biosynthesis. Such specific changes are often associated with alterations in membrane structural components, redox status, and synthesis/utilization of certain amino acids, suggesting that these compounds may achieve differential strain regulation by selectively disrupting these processes [31].

In summary, curcumin and diosmetin regulate LAB metabolism through a dual pattern of “common effects on fundamental pathways + strain-specific differential responses.” The common effects primarily involve amino acid, energy, and nucleotide metabolism, exerting comprehensive influence on core cellular activities. The strain-specific responses reflect selective regulatory mechanisms determined by membrane architecture, antioxidant systems, and the capacity for metabolic network regulation. These findings deepen understanding of the molecular basis underlying interactions between plant-derived bioactive compounds and probiotics, and provide a theoretical foundation for screening natural modulators targeted at specific strains. In food fermentation and probiotic formulation development, such insights can guide the precise application of these compounds to optimize microbial community structure and functionality.

Future research could integrate multi-omics approaches (including transcriptomics, proteomics, and metabolomics) to systematically elucidate the mechanisms of action of plant-derived compounds with varying structures, as well as their metabolic and functional roles in simulated fermentation systems or gut microbial ecosystems. Additionally, studies investigating the influence of dosage, combination strategies, and environmental factors on metabolic regulation plasticity are warranted to achieve more precise probiotic optimization and broader application.

### 4.3. Effects of LAB on Nutritional Quality of C. esculentus Stems and Leaves Silage

The strain used in this study, *Lcb. rhamnosus*, is a homofermentative lactic acid bacterium capable of growth under low pH conditions. Inoculation with LAB can inhibit *Clostridium* and aerobic bacteria, reduce gas production, limit losses of DM and WSC, and decrease proteolysis, thereby improving silage quality [32]. In the present study, the CP content in the *Lcb. rhamnosus* treatment group was significantly higher than in the control, confirming this effect. Elevated NH_3_-N content typically indicates severe amino acid and protein degradation, reflecting poor silage quality [33]. Controlling microbial activity during ensiling is therefore essential to reduce NH_3_-N; proteolysis and amino acid decomposition should be minimized during processing [34]. Our results showed that *Lcb. rhamnosus* reduced CP loss in *C. esculentus* silage, likely through conversion of some proteins into organic acids (e.g., lactic acid), which in turn lowered NH_3_-N levels. The lower NH_3_-N proportion observed in the *Lcb. rhamnosus* group indicates reduced protein degradation and improved silage quality. Furthermore, inoculation significantly reduced NDF contents. NDF and ADF are important indicators of livestock feed intake and digestibility [35]: higher values constrain intake and digestibility because fibers are more difficult to utilize, especially in ruminants where microbial digestion is slow. Lowering NDF and ADF contents can improve palatability and digestibility, enhancing animal growth performance [36].

After 60 days of fermentation, the *Lcb. rhamnosus* group had the lowest pH (4.79), though it remained above the threshold for high-quality silage. Compared with conventional forages, the pH of *C. esculentus* silage declined more slowly and stabilized at a relatively high-level during fermentation [37]. This is likely due to the low soluble sugar content in *C. esculentus*, limiting available substrates for fermentation, thus reducing organic acid production by LAB. Consequently, lactic and acetic acid generation was insufficient to drive further pH decline—a situation similar to substrate limitations reported in alfalfa silage [38]. In addition, certain endogenous compounds in *C. esculentus* straw possess inhibitory effects on LAB growth and metabolism, further constraining acidification efficiency. Nevertheless, LAB inoculation significantly lowered pH and enhanced the fermentation acidification process compared with the uninoculated control. Moreover, introducing endogenous dominant LAB from *C. esculentus* could alleviate such inhibitory effects and promote organic acid formation, offering a feasible strategy for improving silage quality.

Upon opening silage, exposure to air stimulates aerobic microorganism respiration, causing temperature increase and lactic acid decomposition, which raises pH and facilitates the growth of spoilage microbes. Aerobic stability is a key indicator of fermentation quality: shorter stability times are associated with poorer quality, greater susceptibility to aerobic spoilage, and risk of secondary fermentation [39]. LAB enhance aerobic stability by producing lactic acid to lower pH and inhibit spoilage organisms. In this study, the aerobic stability of the *Lcb. rhamnosus* group was significantly greater than that of the control. LAB also exert antioxidant effects, reducing oxidative damage from oxygen exposure and helping preserve nutritional value [40]. Additionally, many LAB produce bacteriocins (e.g., nisin, lactocin, lacticin, enterocin, acidocin, bovicin), which inhibit spoilage bacteria and foodborne pathogens [41].

In conclusion, *Lcb. rhamnosus*, as a homofermentative LAB, effectively lowered pH, reduced CP loss and NH_3_-N levels, and improved fiber fractions (NDF and ADF) in *C. esculentus* silage. Although final pH values after 60 days remained above high-quality silage standards due to low soluble sugar content and potential antimicrobial compounds limiting acid production, LAB inoculation substantially improved aerobic stability. This improvement likely arises from acid production lowering pH, suppression of spoilage bacteria, and bacteriocin generation. The use of endogenous dominant LAB from *C. esculentus* may further alleviate inhibitory effects, stimulate acid production, and enhance overall silage quality.

## 5. Conclusions

This study confirmed that the endogenous active components in *C. esculentus* stems and leaves exert concentration-dependent inhibitory effects on *Leb. buchneri*, *Lpb. plantarum*, and *Lcb. rhamnosus*, with significant differences in tolerance among the strains. Metabolomic analysis indicated that this antibacterial activity is closely associated with coordinated changes in amino acid, energy, and nucleotide metabolism, accompanied by imbalances in membrane structure and redox processes in sensitive strains. Silage experiments demonstrated that inoculation with *Lcb. rhamnosus* effectively lowered pH, reduced CP loss and NH_3_-N accumulation, improved fiber fractions, and enhanced AST. The use of endogenous dominant LAB from *C. esculentus* can alleviate inhibitory effects, promote acid production, and improve silage quality, providing a scientific basis for feed improvement and the precise application of probiotics.

## Figures and Tables

**Table 1:** Morphological characteristics of LAB in *C. esculentus* silage.

Strain	Colonial Morphology	Cell Morphology	Gram Stain Result	Results of the Catalase Test
M1	Large, convex, smooth, opaque, Milky white	Rod-shaped	positive	negative
M2	Small, convex, smooth, translucent, Milky white	Rod-shaped	positive	negative
M3	Medium-sized, convex, smooth, opaque, Grey-white	Short rod-shaped	positive	negative
M4	Large, convex, smooth, opaque, Milky white	Rod-shaped	positive	negative
M5	Small, convex, smooth, opaque, Milky white	Rod-shaped	positive	negative
M6	Small, convex, smooth, translucent, Milky white	Rod-shaped	positive	negative
M7	Large, convex, smooth, translucent, Milky white	Rod-shaped	positive	negative
M8	Medium, convex, smooth, opaque, Grey-white	Short rod-shaped	positive	negative

**Table 2:** The 16S rDNA gene sequencing results of 8 strains of bacteria.

Strain and Number	Compare Ntrain Number	Genetic Sequencing Result	Similarity to the Sequence of the Standard Strain/%
M1	NR113338.1	*Lactiplantibacillus plantarum*	99.72%
M2	NR041293.1	*Lentilactobacillus buchneri*	99.79%
M3	NR113332.1	*Lacticaseibacillus rhamnosus*	100.00%
M4	NR113338.1	*Lactiplantibacillus plantarum*	99.79%
M5	NR041293.1	*Lentilactobacillus buchneri*	99.86%
M6	NR041293.1	*Lentilactobacillus buchneri*	99.79%
M7	NR115605.1	*Lactiplantibacillus plantarum*	99.86%
M8	NR113332.1	*Lacticaseibacillus rhamnosus*	100.00%

**Table 3:** Effects of *Lcb. rhamnosus* addition on the nutritional quality of *C. esculentus* leaf silage.

Items	DM (%)	CP (%)	NDF (%)	ADF (%)	WSC (%)	Ash (%)
CK	28.95 ± 0.15 ^b^	8.29 ± 0.02 ^b^	45.51 ± 0.23 ^a^	26.11 ± 0.24 ^a^	4.17 ± 0.05 ^b^	12.28 ± 0.02 ^a^
*Lcb. rhamnosus*	31.17 ± 0.25 ^a^	8.59 ± 0.05 ^a^	43.91 ± 0.35 ^b^	26.10 ± 0.27 ^a^	5.88 ± 0.05 ^a^	12.31 ± 0.02 ^a^
*t*-test *p*-value	*p* = 0.002	*p* = 0.001	*p* = 0.026	*p* = 0.964	*p* < 0.001	*p* = 0.864

Note: Different lowercase letters indicate significant differences between the *Lpb. plantarum* treatment group and CK (*p* < 0.05). The abbreviations in the figure are defined as follows: DM: dry matter; CP: crude protein; WSC: water-soluble carbohydrates.

**Table 4:** Effects of *Lcb. rhamnosus* addition on the fermentation quality of silage.

Items	pH	LA (g/kg)	AA (g/kg)	NH_3_-N (g/kg)	AST (h)
CK	6.42 ± 0.01 ^a^	0.57 ± 0.05 ^b^	0.27 ± 0.02 ^b^	0.23 ± 0.02 ^a^	72.4 ± 1.8 ^b^
*Lcb. rhamnosus*	4.79 ± 0.13 ^b^	1.32 ± 0.02 ^a^	0.54 ± 0.01 ^a^	0.16 ± 0.01 ^b^	120.5 ± 2.5 ^a^
*t*-test *p*-value	*p* < 0.001	*p* < 0.001	*p* < 0.001	*p* = 0.006	*p* < 0.001

Note: Different lowercase letters indicate significant differences between the *Lpb. plantarum* treatment group and CK (*p* < 0.05). The abbreviations in the figure are defined as follows: LA: lactic acid; AA: acetic acid; NH_3_-N: ammonia nitrogen; AST: aerobic stability time.

## Data Availability

The data presented in this study are available upon request from the first author.
